# Pulse Accumulation Approach Based on Signal Phase Estimation for Doppler Wind Lidar

**DOI:** 10.3390/s24072062

**Published:** 2024-03-23

**Authors:** Naiyuan Liang, Xiaonan Yu, Peng Lin, Shuai Chang, Huijun Zhang, Chen Su, Fengchen Luo, Shoufeng Tong

**Affiliations:** 1 College of Opto-Electronic Engineering, Changchun University of Science and Technology, Changchun 130022, China2023200090@mails.cust.edu.cn (F.L.);; 2 National and Local Joint Engineering Research Center of Space Optoelectronics Technology, Changchun University of Science and Technology, Changchun 130022, China

**Keywords:** coherent Doppler wind lidar, pulse accumulation, phase estimation, cross-correlation

## Abstract

Coherent Doppler wind lidar (CDWL) uses transmitted laser pulses to measure wind velocity distribution. However, the echo signal of CDWL is easily affected by atmospheric turbulence, which can decrease the signal-to-noise ratio (SNR) of lidar. To improve the SNR, this paper proposes a pulse accumulation method based on the cross-correlation function to estimate the phase of the signal. Compared with incoherent pulse accumulation, the proposed method significantly enhances the correlation between signals from different periods to obtain high SNR gains that arise from pulse accumulation. Using simulation, the study evaluates the effectiveness of this phase estimation method and its robustness against noise in algorithms which analyze Doppler frequency shifts. Furthermore, a CDWL is developed for measuring the speed of an indoor motor turntable and the outdoor atmospheric wind field. The phase estimation method yielded SNR gains of 28.18 dB and 32.03 dB for accumulation numbers of 500 and 1500, respectively. The implementation of this method in motor turntable speed measurements demonstrated a significant reduction in speed error—averaging 9.18% lower than that of incoherent accumulation lidar systems. In experiments that measure atmospheric wind fields, the linear fit curve slope between the measured wind speed and the wind speed measured via a commercial wind-measuring lidar can be reduced from 1.146 to 1.093.

## 1. Introduction

Coherent Doppler wind lidar (CDWL) utilizes the Doppler effect to gather information about the wind field. The tool is known for its real-time performance and robust detection capability, finding extensive applications in meteorological research and aviation safety. Nevertheless, during the detection process, due to the interaction between the transmitted signal and the atmospheric aerosol particles, the intensity of lidar echo signal decays rapidly with the extension of detection range, which contributes to a low signal-to-noise ratio (SNR) and complicates the extraction of target motion information [1,2,3,4]. In pulse lidar systems, accumulating multiple pulses over different periods is an important strategy to enhance the SNR. Coherent echo signals can be extracted from strong noise backgrounds using direct accumulation. However, in the coherent Doppler wind lidar system, the initial phase of the pulse signal shows randomness after being modulated by the acousto-optic modulator (AOM), which leads to poor coherence between the transmitted pulse signals, thereby limiting the enhancement in the SNR by direct pulse accumulation.

Various methods have been proposed to improve the SNR and detection precision of the Doppler lidar [5,6,7,8]. Kliebisch et al. proposed a denoising method for CDWL based on convolutional neural networks [9]. Fang et al. introduced a lidar signal-denoising regime based on discrete wavelet transform [10]. Xu et al. put forward a two-stage denoising framework involving singular-value decomposition and adaptive wavelet transform [11]. Zhang et al. used signal segmentation and spectrum averaging to mitigate the impact of noise on weak echo signals, thereby improving the SNR [12]. Frelich et al. suggested estimating the frequency through a coherent accumulation of signals over different periods [13]. Shen et al. established a quantitative relationship between the number of accumulated pulses and improvement in the SNR. Using simulations, Zhang et al. determined that accumulating multiple-period pulses can enhance the SNR; they performed an experimental detection of weak signals in complex environments based on these simulations. Fan et al. proposed using the orthogonal mixing method to construct a phase-detection module, aligning the pulse phases of different periods by shifting the echo signals stored in the oscilloscope along the time axis, thus achieving coherent accumulation after phase alignment. This method has been experimentally proven to enhance the SNR. However, for the pulse accumulation denoising system, the debugging time of non-all-fiber CDWL is limited by its space optical devices. All-fiber CDWL is affected by AOM and other modulators, which introduces pulse incoherence [14,15,16]. At the same time, the quadrature mixing method proposed by Fan et al. has large computation and low real-time performance. This study approaches coherent pulse accumulation by delving into the pulse accumulation challenge in all-fiber CDWL systems and proposing a phase estimation correction method to address the issue of a random initial phase in discrete signals [17,18,19].

This paper initially introduces the system structure of the wind hunter and examines the mathematical model of the lidar echo signal and the SNR model of the system. This proposed method utilizes a cross-correlation function to estimate the phase difference between the impulse signal of different periods and the reference impulse signal. The initial random phases of pulse signals across various periods are rectified using the relevant results, which are selected by calculating the correlation distance between each impulse signal and the reference signal to achieve coherent pulse accumulation in a full-fiber pulsed lidar system. A simulation system for key signal processing technologies of Doppler wind lidar is established, referencing a prototype lidar system parameter. The performance of the phase estimation correction method, the enhancement in SNR gain before and after the application of the method, and the impact of the SNR on the performance of spectrum estimation algorithms are analyzed through simulation. Finally, the speed of an indoor motor turntable and the outdoor atmospheric wind field are measured experimentally to obtain actual data. The effectiveness of our method is demonstrated through both simulations and experiments.

## 2. System Structure and Principles

**Figure 1:**
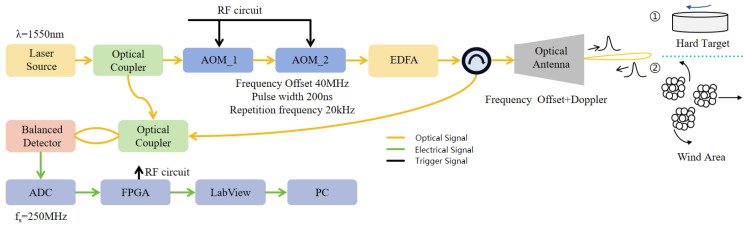
System structure.

The system structure is illustrated in Figure 1.

Let us assume that the seed laser source generates a continuous optical signal with frequency $f_{ss}$, phase $\varphi_{ss}$:

(1) $$S_{t}(t)=\cos(2\pi f_{ss}t+\varphi_{ss}).$$

The laser source has two outputs: a low-power seed laser that is used as a local oscillator (LO), and a power output that is pulsed and frequency-shifted using an AOM. Electronic circuits drive the AOM to shift laser signals by 40 MHz and generate 200 ns Gaussian shaped laser pulses:

(2) $$S_{t}(t)=\sum_{h}Arect(t-hT_{r},\tau )\cos\left\{2\pi (f_{ss}+f_{0})t+\varphi \right\},$$

where $T_{r}$ denotes the pulse repetition period at 10 kHz, h denotes the number of pulses, τ denotes the pulse width at 200 ns, $f_{0}$ denotes the initial frequency offset at 40 MHz, rect(*) denotes the expression of the pulse signal, and φ denotes the initial phase of the pulse signal in each period.

These laser pulses are amplified using an erbium-doped fiber amplifier (EDFA) and then transmitted from port 1 to port 2 of the optical circulator. Laser pulses are transmitted into the atmosphere and aerosol particles scatter the laser signals back into the lens, which in turn are transmitted from port 2 to port 3 of the optical circulator.

The core principle of the CDWL measurement is the Doppler frequency shift caused by the target motion:

(3) $$f_{d}=\frac{2v_{r}}{\lambda },$$

where λ denotes the wavelength at 1550 nm. Therefore, the optical antenna echo signal $S_{r}(t)$ is expressed as

(4) $$S_{r}(t)=\kappa A\sum_{h}rect(t-t_{r}-hT_{r},\tau )\cos\left\{2\pi (f_{ss}+f_{0}+f_{d})t+\varphi -\varphi_{0}\right\},$$

where κ denotes the attenuation of link, $t_{r}$ denotes the time delay caused by the signal traveling the distance, and $\varphi_{0}$ denotes the phase delay caused by the distance.

Backscattered and LO signals are optically mixed using an optical coupler. Optically mixed signals are heterodyne-detected using an optical balanced detector, which generates RF signals:

(5) $$S_{r}(t)=u_{r}\sum_{h}rect(t-t_{r}-hT_{r},\tau )\cos\left\{2\pi (f_{0}+f_{d})t-\varphi_{0}\right\},$$

where $u_{r}$ denotes the amplitude of the signal through the optical balance detector. The echo signal of the pulse lidar is then expressed as $R(t)=S_{r}(t)+N(t)$, where N(t) represents the independent Gaussian white noise in each pulse period. These RF signals are acquired at a 250 MHz sampling rate using an analog-to-digital converter card (ADC). Sampling the echo signal R(t) at a sampling frequency $f_{s}$ over time $T=M/f_{s}$ results in the discrete signal sequence r(m):

(6) $$r(m)=B\cos\left\{2\pi (f_{0}+f_{d})m/f_{s}+\varphi \right\}+N(m),m=0,1\ldots ,M-1,$$

where $B\cos(2\pi f_{d}m/f_{s}+\varphi )$ denotes the discretized S(t) signal after sampling, B refers to $u_{r}rect(t-t_{r}-hT_{r})$, $f_{d}$ denotes the Doppler frequency shift, and N(m) denotes the discrete noise of the echo signal in each period.

The discrete echo pulse signals in adjacent pulse periods $r_{1}(m)$, $r_{2}(m)$ can be expressed as

(7) $$r_{1}(m)=B_{1}\cos\left\{2\pi (f_{0}+f_{d})m/f_{s}+\varphi_{1}\right\}+N_{1}(m),m=0,1\ldots ,M-1$$

(8) $$r_{2}(m)=B_{2}\cos\left\{2\pi (f_{0}+f_{d})m/f_{s}+\varphi_{2}\right\}+N_{2}(m),m=0,1\ldots ,M-1,$$

where $B_{1}$ and $B_{2}$ denote the amplitudes of the pulse signals in adjacent periods; $\varphi_{1}$ and $\varphi_{2}$ denote the initial phases of the pulse signals in adjacent periods; and $N_{1}(m)$ and $N_{2}(m)$ denote the discrete noises of the signals in adjacent periods.

The accumulation process for two adjacent pulse period signals can be expressed as

(9) $$r_{1}(m)+r_{2}(m)=B_{1}B_{2}\cos\left\{2\pi (f_{0}+f_{d})m/f_{s}\right\}\cos(\frac{\left|\varphi_{1}-\varphi_{2}\right|}{2})+\sum_{i=1}^{2}N_{i}(m),$$

where $B_{1}B_{2}\cos\left\{2\pi (f_{0}+f_{d})m/f_{s}\right\}$ represents the signal part after pulse accumulation. In the laser wind lidar system, the initial phase of signals from different periods is random, denoted as $\varphi_{1}\neq \varphi_{2}$, due to which the signal gain in $\sum r_{i}(m)$ cannot reach its maximum. Using phase correction, consistency can be obtained for phases of signals from different periods, that is, $\varphi_{1}=\varphi_{2}$, which implies that the signals have coherence, and at this time, the signal gain in $\sum r_{i}(m)$ reaches its maximum value.

To achieve phase correction within a non-fully coherent pulse lidar system, a correction scheme grounded in signal phase estimation is proposed. After performing analog-to-digital conversion on the echo signal, the phase discrepancy between each period pulse signal and the reference pulse signal is derived using the cross-correlation function. These findings are utilized to adjust the signal of each period such that its phase converges toward 0 rad in relation to the reference signal. This alignment ensures coherence among all period signals throughout the accumulation process, facilitating a shift from non-coherent pulse accumulation to coherent pulse accumulation.

For two reference pulse signals of M points, $r_{0}(m)$ and any period pulse signal $r_{x}(m)$, their cross-correlation function is sought. The expression is given by

(10) $$r_{0x}(l)=\sum_{l=1-m}^{m-1}r_{0}(m)r_{x}(m+l),$$

where l represents the time delay on the discrete time axis. The two discrete pulse signals exhibit the smallest phase difference at the time delay $l^{\prime }$ corresponding to the peak value of their cross-correlation function results. In a lidar system, when dealing with two pulse period signals with distinct phases, the phase adjustment on the time axis of one signal is determined by shifting it left or right based on the outcomes of their cross-correlation function, thereby bringing the phases of the two period signals into close alignment. For a 40 MHz sine signal sampled at 250 MHz, an average period corresponds to 6.25 points, resulting in a phase alignment precision of 0.497 rad with this algorithm.

After conducting a fast Fourier transform (FFT) on the accumulated signal, the obtained discrete spectrum I(h) can be interpolated and corrected to yield the accurate frequency, as follows: the actual frequency $f=f_{0}+f_{d}$ lies between the spectral lines. Hence, it is, respectively, represented as the integer part h and the fractional part δ(−0.5≤δ≤0.5). The spectral line *h* is the spectral peak of the amplitude spectrum and δ is the frequency correction amount, which can be represented by the amplitude of the spectral peak line and the two adjacent spectral lines on its left and right.

(11) $$\delta =\frac{\left|I(h+1)\right|+\left|I(h-1)\right|}{2\left|I(h)\right|+\left|\left|I(h+1)\right|-\left|I(h-1)\right|\right|}$$

Wind speed can be derived from the frequency shift amount $f_{d}=h+\delta -f_{0}$:

(12) $$v_{r}=\frac{1}{2}f_{d}\lambda .$$

The SNR of the echo signal in a single pulse period can be expressed as

(13) $$SNR=\frac{{\left[S_{r}(t)\right]}^{2}}{E{\left[N(t)\right]}^{2}}.$$

## 3. Pulse Accumulation Based on Cross-Correlation Function for Phase Estimation Simulation Experiment

The essential signal processing technology simulation system for the Doppler wind lidar relies on the LabView2018 software platform for simulation analysis. The simulation parameters are referenced from the actual system, with the parameters of the transmitted pulse signal set as follows: a fundamental frequency shift of 40 MHz, a sampling frequency of 250 MHz, a pulse width of 200 ns, and a repetition frequency of 20 kHz.

**Figure 2:**
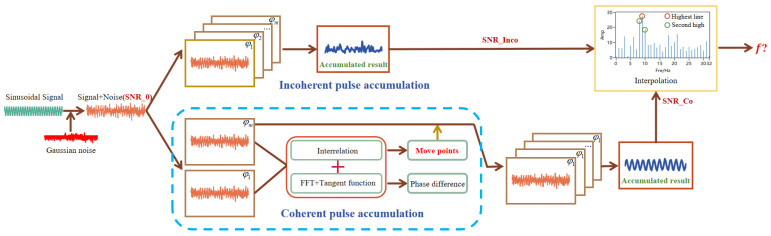
Simulation experiment.

The simulation experiment process is illustrated in Figure 2. The blue dashed box represents the phase correction algorithm, where the reference pulse signal is cross-correlated with other period signals, and the phases of the other signals are adjusted to be consistent with the reference signal to ensure coherence among all pulse signals during the accumulation process [20,21,22]. Meanwhile, a discrete single-frequency reference sinusoidal pulse signal is established along with other sinusoidal pulse signals featuring random initial phases. The initial phase of these additional sinusoidal signals is randomly distributed within the range of (−π~π). Gaussian white noise with a specified power level is added to these signals, setting the initial SNR at −13 dB. The non-coherent pulse accumulation result is obtained by directly accumulating these signals in the time domain. Coherent pulse accumulation is used to obtain the phase difference between the initial random phase pulse signal and the reference signal after passing through the phase estimation module, and then moved left and right in the time domain according to the result to achieve phase alignment. The cumulative result is obtained by accumulating these aligned signals in the time domain. The SNR gains of the two methods are calculated for different accumulation counts. The improvement in the SNR can enhance the lidar detection precision; therefore, the spectrum interpolation algorithm for analyzing the frequency shifts of the wind-measuring lidar is simulated for its noise resistance performance. The SNR of the pulse signal is set to vary from −13 dB to −20 dB based on the precision of wind speed analysis at 0.2 m/s. Using Δf=2Δv/λ, it can be deduced that the frequency analysis precision should be 0.258 MHz; thus, the frequency of the pulse signal is randomly set within the interval (40.258 MHz, 39.742 MHz). For each SNR condition, 10,000 sets of pulse signals are generated, and the standard deviation (STD) of the 10,000 frequency spectrum interpolation algorithm analysis frequencies is statistically analyzed. The overall simulation results are as follows.

**Figure 3:**
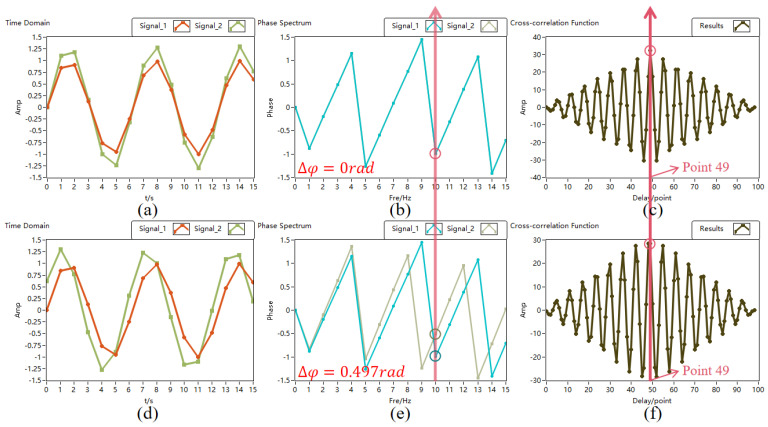
Two signals with the same phase and adjustable phase accuracy using the method discussed in the text. (**a**) In-phase sinusoidal signal time domain graph. (**b**) In-phase sinusoidal signal phase spectrum. (**c**) Cross-correlation result of the in-phase sinusoidal signal. (**d**) Phase difference 0.497 rad sinusoidal signal time domain graph. (**e**) Phase difference 0.497 rad sinusoidal signal phase spectrum. (**f**) Cross-correlation result of the phase difference 0.497 rad sinusoidal signal.

Figure 3 shows the time domain graphs, cross-correlation function outcomes, and phase spectra of two sinusoidal signals. To simulate echo signals from distinct periods, two discrete sinusoidal signals with differing amplitudes, matching frequencies, and identical initial phases are depicted in Figure 3a. In the phase spectrum of Figure 3b, the two signals align precisely at the 10th spectral line corresponding to 40 MHz, demonstrating phase consistency. Additionally, the peak of the cross-correlation function outcomes of the two signals shown in Figure 3c occurs at the center, specifically at the 49th point. This implies that at various time delays, the two in-phase signals can achieve maximum similarity overlap when they are centered at the time delay. In Section 2, we analyzed the phase precision adjusted using this algorithm to be 0.497 rad, as indicated in Figure 3d, with the initial phase difference between the two signals set to 0.497 rad. The phase spectra and cross-correlation function results of the two are depicted in Figure 3e,f, revealing a 0.497 rad disparity at the 10th spectral line, while the peak of the cross-correlation outcome remains centered at the 49th point. These findings suggest that the phase precision identifiable and correctable by the algorithm proposed in this study aligns with the theoretical calculation values. Subsequently, a phase correction simulation experiment was conducted on two sinusoidal signals in the lidar system with a random phase difference of (−π~π), and the results are outlined below.

**Figure 4:**
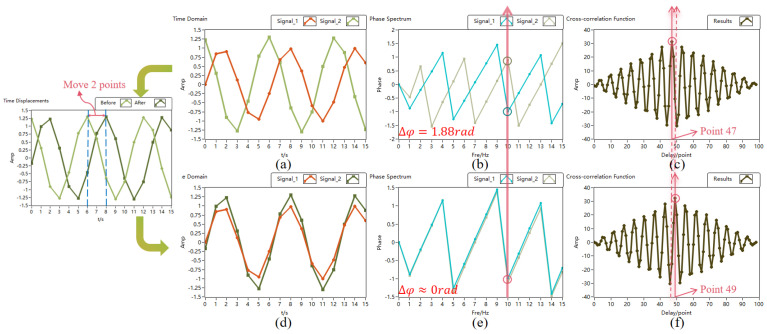
Phase correction alignment process diagram. Before adjusting (**a**) the time domain graph; (**b**) the phase spectrum; (**c**) the cross-correlation result. After adjustment of (**d**) the phase spectrum; (**e**) the time domain graph; (**f**) the phase spectrum.

Figure 4 illustrates the process of phase correction alignment for two sinusoidal signals with different phases. Two sinusoidal signals with a phase difference of 1.9 rad are shown in Figure 4a. In Figure 4b,c, a clear phase difference can be observed in the phase spectrum of the two signals, and the peak of the cross-correlation function shifts away from the center point, appearing at the 47th point. This indicates the difference in the peak position from the in-phase sinusoidal signal cross-correlation result peak, suggesting a phase difference between the signals. Following the point difference on the time axis, one of the signals is shifted, as shown in Figure 4d. After adjustment, as shown in Figure 4f, the peak of the cross-correlation function result of the two signals returns to the center, at the 49th point, and in the phase spectrum of Figure 4e, the amplitudes of the 10th spectral line of the two signals almost coincide. The algorithm adjustment aligns the phases of the two signals, fulfilling the requirement for coherent accumulation. In the pulse accumulation process of the lidar system, each period signal can undergo phase estimation correction with the reference pulse signal to achieve long-term coherent pulse accumulation. A simulation experiment is devised, and the SNR gains of in-phase pulse accumulation, non-in-phase pulse accumulation, and phase estimation-corrected non-in-phase pulse accumulation for different accumulation counts are statistically analyzed, yielding the following results.

**Figure 5:**
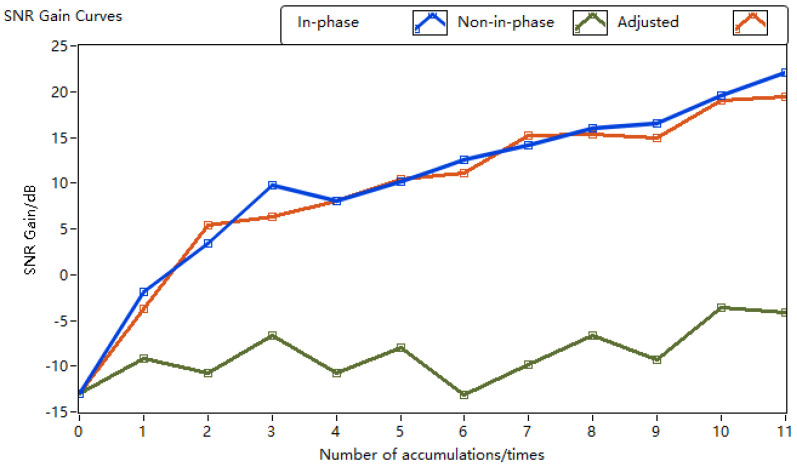
Pulse accumulation signal-to-noise ratio gain curves.

Figure 5 depicts the SNR gain curves for three types of pulse accumulation: in-phase pulse, non-in-phase pulse, and adjusted non-in-phase pulse. It is evident that the initial SNR for all three pulse accumulation result curves is −13 dB. With an increase in the number of accumulations, the SNR gain of in-phase pulse accumulation notably increases, achieving a 30 dB enhancement in SNR at 2000 accumulations, while that of random phase pulse accumulation experiences an 8 dB enhancement in the SNR. This suggests that coherent pulse accumulation can yield a higher SNR gain than non-coherent pulse accumulation for the same number of accumulations. From the blue and green curves, it can be observed that after applying the phase estimation correction algorithm, the SNR gain of random phase pulse accumulation aligns closely with that of in-phase pulse accumulation. The simulation experiment validates that the phase estimation correction algorithm effectively enhances the correlation between pulse signals with different initial phases, ensuring that the SNR gain after pulse accumulation is within the same order of magnitude as that of in-phase pulse accumulation.

After FFT spectrum conversion, the spectral resolution is not high enough due to spectrum leakage and fence effect. In this paper, a spectral correction algorithm is introduced to improve the accuracy of analytic frequency. Since the two pulse accumulation methods have different gain effects on the SNR, the necessity of improving SNR is reflected by evaluating the anti-noise performance of the spectrum correction algorithm.

**Figure 6:**
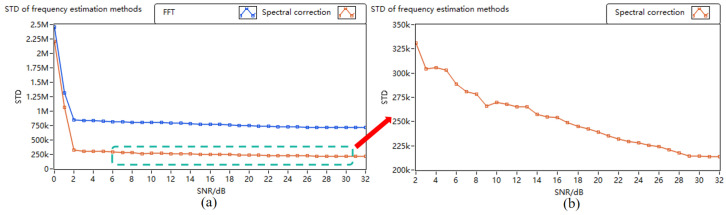
Anti-noise performance of discrete spectrum interpolation correction method. (**a**) All curves; (**b**) local curve of interpolation correction method.

Figure 6 illustrates the noise resistance performance curves of two methods for analyzing frequency through discrete spectrum. The blue curve represents the standard deviation curve of the frequency analysis method obtained following the FFT time–frequency transformation of the discrete spectrum, while the pink curve depicts the standard deviation of the corrected frequency obtained after further spectral line interpolation of the discrete spectrum. Evidently, for SNR values lower than −1 dB, the frequency standard deviations of both algorithms considerably exceed the anticipated precision requirement; for SNR = −1 dB and higher, the standard deviations of both frequency estimation methods exhibit minimal change. The FFT-only algorithm maintains a frequency standard deviation within the range of 750 kHz–1 MHz, corresponding to a speed measurement precision of 0.58–0.78 m/s. Conversely, the standard deviation of the corrected frequency after spectral line interpolation of the discrete spectrum remains in the range of 200–300 kHz, corresponding to a speed measurement precision of 0.16–0.23 m/s. The simulation experiment demonstrates that a high SNR can enhance the precision of frequency analysis; when the SNR remains constant, the noise resistance performance of the system improves after correction through the spectral line interpolation algorithm compared to FFT-only spectrum conversion.

## 4. Lidar Prototype Speed Measurement Experiment

### 4.1. Motor Turntable Speed Measurement Experiment

**Figure 7:**
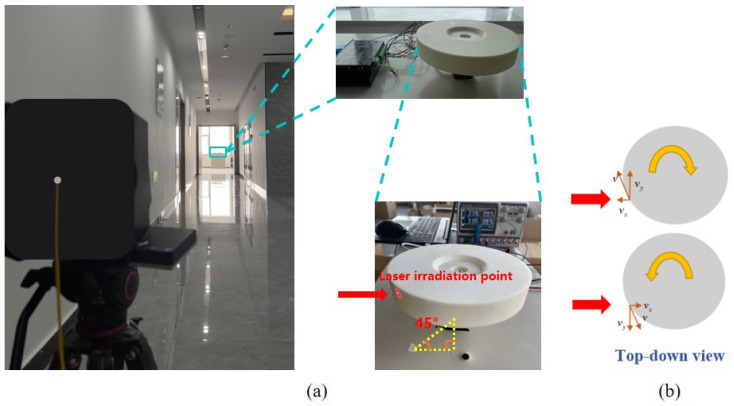
Experimental setup. (**a**) Lidar with motor and turntable; (**b**) top view of the laser irradiating the turntable.

A lidar prototype was assembled based on the theory of phase estimation and correction using cross-correlation functions to measure the speed of a turntable controlled by a variable frequency drive motor, as depicted in Figure 7. The test bench, featuring the variable speed motor, was positioned 45 m away from the lidar, which emits pulse laser irradiating the circular turntable connected to the motor shaft, as illustrated in Figure 7a. By adjusting the motor speed, the speed of the turntable measured by the Doppler wind lidar prototype was compared before and after implementing the phase estimation correction algorithm at the same accumulation count, which helped determine the speed measurement accuracy of the lidar prototype.

Moreover, to replicate radial direction wind speed changes while moving away from and approaching the lidar fully, the upper computer controlled the motor to drive the turntable to rotate clockwise and counterclockwise during the experiment. As illustrated in Figure 7b, when the turntable rotates clockwise, the linear speed at the laser irradiation point on the turntable plane exhibits a radial component moving towards the lidar, resulting in a positive measured speed. Conversely, when the turntable rotates counterclockwise, the radial component of the speed at the laser irradiation point moves away from the lidar, yielding a negative measured speed. The experimental results are summarized as follows.

**Figure 8:**
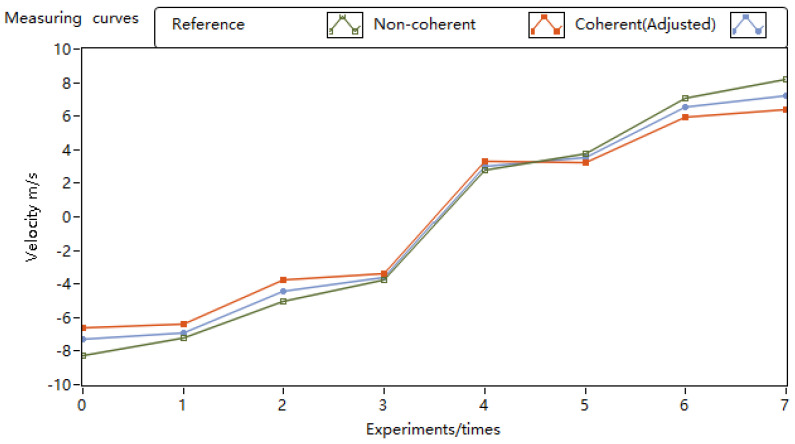
Motor turntable speed measurement experiment velocity curves.

Figure 8 displays the motor turntable speed curves obtained using different pulse accumulation methods. Throughout the experiment, the turntable speed was controlled to vary from −8 m/s to 8 m/s. The curves for non-coherent accumulation and coherent accumulation represent the speed curves measured by the lidar prototype before and after applying the phase estimation correction algorithm.

At lower speeds, the fit among the three curves was high. However, as the speed gradually increased, the results of the coherent pulse accumulation, after the application of the phase estimation correction algorithm, exhibited fewer errors with the actual set turntable speed than the speed measured by the non-coherent pulse accumulation of the lidar prototype.

In the motor turntable experiment, the hard target turntable was 45 m away from the lidar, which resulted in an improved SNR of the hard target reflection in the echo signal. Therefore, the speed advantage measured by coherent accumulation is not obvious compared with that measured by incoherent accumulation. Moreover, to better evaluate the precision improvement before and after applying the algorithm, four turntable speeds with high curve fitting degrees were selected, from –5 m/s to 3.74 m/s, and multiple speed measurement experiments were conducted to analyze the mean squared error (MSE). At the same time, the SNR gain effects of coherent and incoherent methods were analyzed in four different speed measurement experiments when the accumulation number was 50, and power spectral density (PSD) changes after coherent and non-coherent accumulation are given for the speed of 3.74 m/s. The results are summarized as follows.

**Figure 9:**
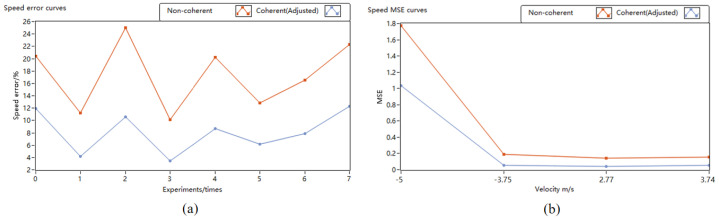
Motor turntable speed measurement experiment error curves. (**a**) Error curve; (**b**) mean squared error (MSE) curve.

The speed measurement experiment error curve is depicted in Figure 9. In Figure 9a, it is evident that at turntable speeds of −8.92 m/s, −7.21 m/s, −5 m/s, −3.75 m/s, 2.77 m/s, 3.74 m/s, 7.08 m/s, and 8.2 m/s, the speed measurement error of the system—after the application of the phase estimation correction algorithm—was reduced by 8.4%, 7.1%, 14.4%, 6.7%, 11.6%, 6.7%, 8.6%, and 10%, respectively, compared to the non-coherent all-fiber lidar prototype system, indicating a significant reduction in error. Furthermore, for speeds corresponding to significant error reduction, namely −5 m/s, −3.75 m/s, 2.77 m/s, and 3.74 m/s, eight independent experiments were conducted to statistically analyze the MSE of speed measurement, as illustrated in Figure 9b. At an approximate absolute speed of 3 m/s, the MSE before and after applying the phase estimation correction algorithm remained relatively stable, averaging a reduction of 0.11. The experimental results conclusively demonstrate that the application of this phase estimation correction algorithm effectively improves the precision of lidar speed measurement.

**Figure 10:**
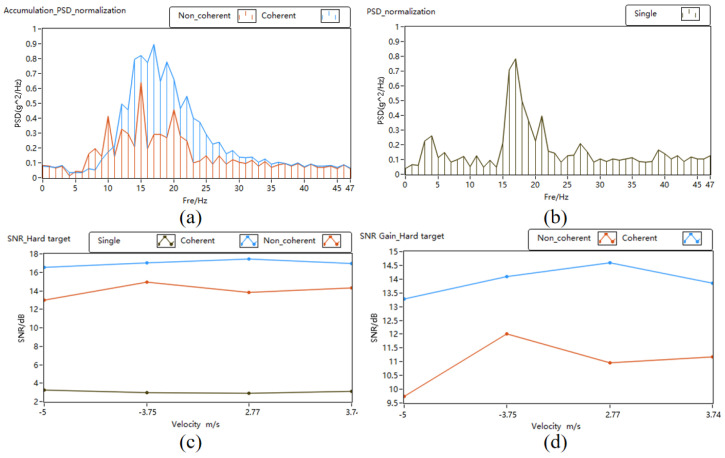
PSD, SNR, and SNR gain of pulse accumulation. (**a**) PSD of pulse accumulation; (**b**) PSD of single pulse; (**c**) SNR; (**d**) SNR gain.

The PSD, SNR, and SNR gain of pulse accumulation is depicted in Figure 10. In Figure 10a,b, it is evident that the signal power increases with the accumulation times, and the noise power is suppressed in the normalized PSD. At the same time, compared with the non-coherent accumulation, the signal power of the coherent accumulation is greater. In Figure 10c,d, the signal-to-noise ratio is improved after the pulse accumulation in the four different speed measurement experiments. The signal-to-noise ratio gains of the coherent accumulation are 13.28 dB, 14.1 dB, 14.59 dB, and 13.86 dB, respectively. The signal-to-noise ratio gains of the non-coherent accumulation are 9.74 dB, 12.02 dB, 10.95 dB, and 11.18 dB, respectively. The experimental results show that the application of the phase estimation correction algorithm effectively improves the signal-to-noise ratio gain.

### 4.2. Wind Field Wind Speed Measurement Experiment

**Figure 11:**
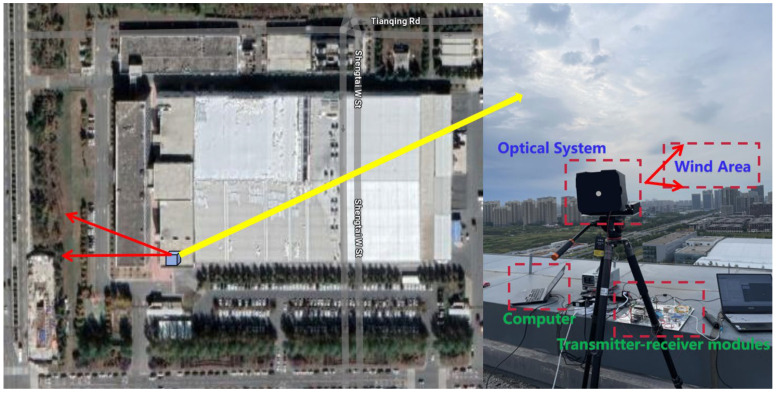
Wind-measuring lidar prototype and experimental site.

After verifying motor turntable speed measurement performance of the wind-measuring lidar prototype, an experiment aimed at measuring the actual atmospheric wind speed was conducted. In mid-October of 2023, the soft target wind field wind speed measurement experiment was completed, with the experimental setup depicted in Figure 11.

The actual system echo signal is susceptible to interference from external environmental factors, as well as system dark current, thermal noise, etc., rendering its spectral components complex and not directly deducible from the power spectral density (PSD) for SNR. Therefore, the scattering of laser signals in the air results in weaker echo signals for further range gates of the measured signal. The lidar prototype in this study contained power calculations for 10 range gates, specifically for range gates 1 to 9, and the 14th range gate. The 14th range gate is considered background noise, while the peak parameter of the 9th range gate after calculation is regarded as dynamic noise. The SNR calculation formula is as follows:

(14) $$SNR=10\log\frac{P_{s}}{P_{n}},$$

where $P_{s}$ denotes the peak power of the current group after subtracting background noise, and $P_{n}$ denotes the peak power of the 11th group after subtracting background noise. To reduce errors, the power of the signal and noise were averaged based on the current number of accumulations when the single-period SNR was calculated. The SNR gains under different accumulation counts are as follows.

**Figure 12:**
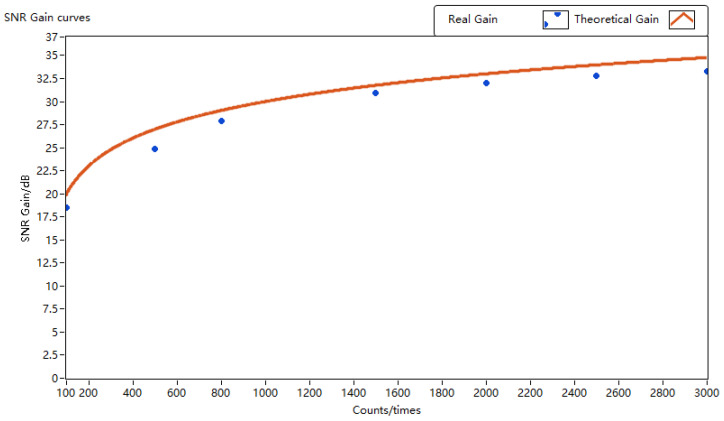
Actual SNR gain vs. theoretical SNR gain curve.

Table 1 lists changes in the SNR of the signal from fiber reflection with an increasing number of accumulations. Throughout the statistical process, as the number of accumulations varied, the average single-period SNR remained at approximately 29 dB, indicating the relative stability of the system signal at this experimental stage. The actual SNR gain alterations with the number of accumulations are depicted in Figure 12. Notably, after the application of the phase estimation correction algorithm, the actual system SNR gain demonstrated a clear increasing trend with the number of accumulations, reaching 24.83 dB, 30.89 dB, and 32.04 dB at accumulation counts of 500, 1500, and 2000, respectively. This underscores the ability of the lidar system to achieve effects similar to the SNR gain of coherent pulse accumulation using this algorithm.

By continuously monitoring the wind field through the wind-measuring lidar prototype, echo signal data samples measuring 2500 pulses were collected at the 120 m range gate of the echo signal, and two types of pulse accumulation were conducted, yielding the following results.

**Figure 13:**
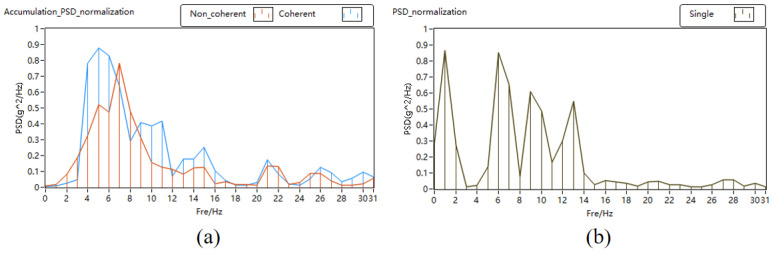
PSD of lidar-measured pulse accumulation. (**a**) PSD of pulse accumulation; (**b**) PSD of single pulse.

Figure 13 shows the signal PSD diagram of the pulse accumulation of the lidar echo signal at the distance gate of 120 m. In Figure 13a,b, it is evident that the signal power increases with the accumulation times, and the noise power is suppressed in the normalized PSD. Figure 13b shows the PSD of the single-cycle echo signal, and the weak signal is submerged in the noise, so effective information cannot be obtained from the PSD. After 2500 times of pulse accumulation, as shown in Figure 13a, there are obvious spectral peaks in the PSD. After accumulation, the accurate wind speed can be obtained. Compared with the incoherent pulse accumulation, the power ratio of the coherent pulse accumulation signal is higher. However, the improvement effect of the coherent and incoherent pulse accumulation on the signal SNR gain is relatively weak, which is due to the destruction of the phase of the atmospheric echo signal in the propagation process, which interferes with the effect of pulse accumulation.

Meanwhile, 700 wind speed data samples were collected at the 120 m range gate of the echo signal and fitted with calibrated wind speed samples, which were measured using a commercial wind-measuring lidar. The parameters of commercial wind lidar are shown in Table 2, yielding the following results.

**Figure 14:**
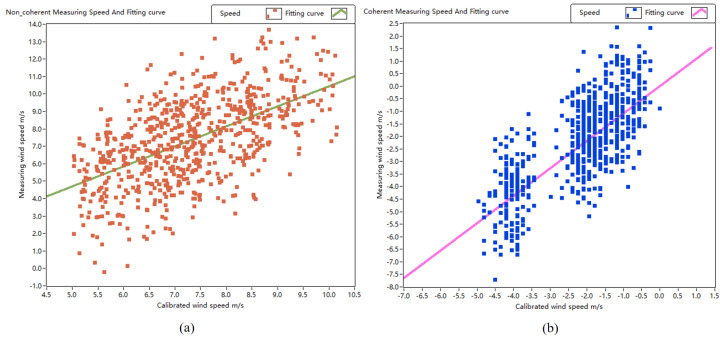
Fitting of lidar-measured speed vs. calibrated speed. (**a**) Non coherent; (**b**) coherent.

Figure 14 displays the fitting curve of 700 sample wind speeds measured simultaneously using the lidar prototype and the commercially calibrated lidar. The *x*-axis represents the calibrated wind speed measured using the commercial wind-measuring lidar, while the *y*-axis represents the wind speed measured using the lidar prototype at the same time. The wind speed measured before the phase estimation correction of the lidar prototype pulse signal is depicted in Figure 14a, and the fitting curve of the two data sample sets obtained by linear fitting is y=1.146x−1.032. Subsequently, the lidar prototype-measured wind speed after the phase estimation correction is illustrated in Figure 14b, and the fitting curve of the two data sample sets is y=1.093x+0.205. Upon comparing the fitting curves before and after adjustment, it is observed that the fitting slope decreased from 1.146 to 1.093, approaching 1. These fitting results indicate that the accuracy of the wind-measuring lidar prototype improved after adjustment, and the prototype exhibited good consistency with the commercially calibrated lidar in measuring wind speed.

## 5. Conclusions

This study aims to enhance the pulse accumulation SNR gain of all-fiber pulsed lidar systems. The efficacy of the proposed method in mitigating the incoherence among pulses of various periods is evaluated. Furthermore, integrating this method with the discrete spectrum interpolation algorithm can enhance the measurement precision of lidar systems, especially in high-background-noise environments. To validate the proposed solution, a Doppler coherent lidar prototype was constructed. The experimental results indicate that after adjustments using the phase estimation method, the simulated experiment SNR gains at accumulation counts of 500, 1500, and 2000 can reach 28.18 dB, 32.03 dB, and 32.44 dB, respectively. Similarly, the actual lidar system SNR gains can reach 24.83 dB, 30.89 dB, and 32.04 dB, respectively. Furthermore, the linear fitting slope of the measured wind speed versus the commercially calibrated lidar wind speed can be reduced from 1.146 to 1.093. In the motor turntable speed measurement experiment, the error in the measured speed of the turntable is reduced by an average of 9.18% after applying the method. These results demonstrate the effectiveness of the proposed method for the all-fiber pulsed lidar system, as it enhances the correlation of echo signals from different periods, boosts the SNR gain of weak signal pulse accumulation, and achieves a high-precision speed-measuring lidar system.

## Figures and Tables

**Table 1:** SNR gains at different accumulation counts.

Counts	100	500	800	1500	2000	2500	3000
Single SNR	29.65 dB	29.58 dB	29.76 dB	29.97 dB	30.18 dB	29.84 dB	29.67 dB
Total SNR	48.19 dB	54.41 dB	57.68 dB	60.86 dB	62.22 dB	62.62 dB	62.99 dB
Real Gain	18.54 dB	24.83 dB	27.92 dB	30.89 dB	32.04 dB	32.78 dB	33.32 dB
Theo-Gain	20.00 dB	26.99 dB	29.03 dB	31.76 dB	33.01 dB	33.98 dB	34.77 dB

**Table 2:** Details of the commercial wind lidar.

Parameters	Value
Distance	50–200 m
Section number	10
Measurement rate	4 Hz
Wind speed accuracy	0.1 m/s
Wind speed range	0–50 m/s
Wind direction range	−90–90°
Wind direction accuracy	0.5°

## Data Availability

The data presented in this study are available on request from the corresponding author. The data are not publicly available due to privacy.
